# The impact of 2 weeks wait referral on survival of head and neck cancer patients

**DOI:** 10.1007/s00405-023-08152-0

**Published:** 2023-08-01

**Authors:** Aleix Rovira, Beth Russell, Priyanka Trivedi, Onaiho Ojo, Richard Oakley, Edie Byrne, Avisha Daryanani, Mieke Van Hemelrijck, Ricard Simo

**Affiliations:** 1 Division of Surgical Oncology, Head and Neck Surgery, Guy’s and St Thomas’ NHS Foundation Trust, London, UK; 2 Cancer Epidemiology, King’s College London, London, UK; 3 King’s College London, London, UK; 4 Translational Oncology and Urology Research, King’s College London, London, UK

**Keywords:** Head and neck cancer, Hospital referrals, Diagnosis, Early diagnosis of cancer, Cancer screening, Survival

## Abstract

**Purpose:**

This study aims to evaluate the association between 2 weeks wait referral and survival in the head and neck cancer.

**Methods:**

Retrospective cohort study of consecutively discussed new head and neck cancer patients at large United Kingdom Cancer Alliance including two tertiary referral hospitals and two district general hospital.

**Results:**

A total of 276 cancer patients were included for analysis. Patients referred under the 2 weeks wait had were seen and diagnosed sooner from referral (*p* < 0.0001 and *p* < 0.0001 respectively). However, this did not translate into better survival outcomes. No survival differences were seen between those patients that were managed within the proposed cancer targets and those that were not.

**Conclusions:**

The 2 weeks wait head and neck cancer pathway did not offer a survival advantage. Targeting the delay in referral as well as delay in treatment to prevent late-stage cancer presentation is paramount. Fulfilment of cancer time targets do not translate into better outcomes and should not be prioritised to clinical judgement.

## Introduction

Head and neck cancer (HNC) is the 8th most common cancer in the UK and its incidence is increasing [1]. Care for HNC patients has been centralised from 2007 and patients are treated in a limited number of hospitals, therefore referral plays a significant role in the diagnosis, treatment, and survival outcomes. The 2WW pathway ensures that general practitioners (GP) can urgently refer patients suspected to have cancer to be seen within 14 days in a specialist clinic. The rationale for it was established after the EUROCARE studies in 1995 revealed that treatment outcomes for cancers, including HNC, are worse in the UK compared to other European countries [2–4]. Even though the conclusions obtained from these studies could be challenged [5], the UK government proposed the 2WW referral pathway in 1998 to improve patient outcomes in response to this finding, and it was subsequently implemented in 2000 [6]. The National Institute for Health and Care Excellence (NICE) published national referral guidelines in 2005, setting out “red flag” signs and symptoms of HNC that would require a patient to be referred urgently to a specialist [7]. These guidelines have since been reviewed and were last updated in 2015 [8]. Published literature has yet to comprehensively assess the effect of the 2WW pathway on patient outcomes. There is published data assessing the diagnostic yield of the 2WW pathway; establishing that 2WW does not have an association with a diagnosis of early-stage disease [9]. However, there is limited research to establish the effect of the 2WW on the survival of patients.

This paper aims to assess the impact of the 2WW HNC on survival outcomes compared to other referral pathways. As a secondary outcome, we analysed the impact of fulfilment of stablished time targets into survival outcomes.

## Materials and methods

This is a retrospective cohort study of all HNC patients discussed at weekly multi-disciplinary team (MDT) meetings within the South East London Head and Neck Cancer Network from January to October 2017. STROBE statement checklist was followed for the creating of this manuscript. Electronic patient records (EPR) were used to cross-check the cancer diagnosis with pathology reports and clinic letters (referral letters, outpatient clinic letters, MDT outcome forms) to ensure accuracy Information was extracted from patient and histopathology reports. Exclusion criteria included previous history of HNC, tumour location in thyroid, skin or eye, benign pathology or patients with incomplete data available. A database was compiled with patient referral route, presenting complaint, time from referral to the first appointment with a specialist and time to diagnosis. The staging system used was the American Joint Committee on Cancer TNM staging system, 7th edition [10]. To assess survival, time from diagnosis to final follow up or death was collected. Demographic information including age, smoking status and alcohol consumption was also collected.

Descriptive statistics were conducted using Student *t* tests to compare continuous data and *X*^2^ for categorical data. The proportion of patients diagnosed at early versus late stage, and with curative versus palliative/best supportive care between the different referral pathways were compared using *X*^2^. Kaplan–Meier curves and Log-Rank tests were used to compare 5 years overall survival (OS) and recurrence-free survival (RFS) from date of diagnosis confirmed by biopsy. Recurrence was defined as histologically confirmed or as per MDT consensus. Patients were censored at either death, their last follow up appointment or 5 years after diagnosis. All statistical analyses were conducted using STATA/MP 17.0.

Using the NHS Health Research Authority decision tool this project was determined to be a service evaluation thereby not requiring ethical approval. The project was approved with reference number 11663 at Guy’s and St Thomas’ NHS Foundation Trust. Principles of Good Clinical Practice were adhered to throughout the study.

## Results

During the studied period, 396 HNC patients were discussed in the regional head and neck MDT, excluding thyroid cancer. A total of 276 patients were included for final analysis after exclusion of patients according to the described exclusion criteria. The cohort characteristics and tumour stage and location according to referral method are described in Table 1. There was no difference between patients presenting through and outside the 2WW pathway apart from a larger percentage of males (*p* = 0.029) and current smokers (*p* = 0.009) in the 2WW group. There was a higher proportion of patients diagnosed at a late stage (77.3%) in patients referred under the 2WW compared to those referred via other pathways (63.2%) (*p* = 0.012) (Table 2). Only patients diagnosed through the emergency department (95%) and other 2WW pathways (80%) had higher percentages of advanced stage at diagnosis. However, there was not a significant difference in the intention to treat (curative vs palliative) between the referral pathways (*p* = 0.184) (Table 2).

**Table 1:** Cohort characteristics and tumour stage and location according to referral method

Age	Total		H&N 2WW		Other referral pathway		*p* value
	*n*	(%)	*n*	(%)	*n*	(%)	
< 50	36	13.00	22	12.20	14	14.70	0.364
50–59	75	27.20	53	29.30	22	23.20	
60–69	85	30.80	61	33.70	24	25.30	
70–79	55	19.90	30	16.60	25	26.30	
> = 80	25	9.10	15	8.30	10	10.50	
Mean (SD)	62.68 (11.78)		62.20 (11.18)		63.56 (12.88)		
Sex							
Female	79	28.60	44	24.30	35	36.80	**0.029**
Male	197	71.40	137	75.70	60	63.20	
Smoking status							
Current smoker	100	36.20	76	42.00	24	25.30	**0.009**
Ex-smoker	85	30.80	55	30.40	30	31.60	
Non-smoker	91	33.00	50	27.60	41	43.20	
Stage at diagnosis							
I	46	16.70	21	11.60	25	26.30	**0.020**
II	30	10.90	20	11.00	10	10.50	
III	33	12.00	23	12.70	10	10.50	
IV	167	60.50	117	64.60	50	52.60	
Tumour location							
Hypopharynx/cervical oesophagus	15	5.40	8	4.40	7	7.40	**0.011**
Ear	4	1.40	0	0.00	4	4.20	
Larynx	44	15.90	31	17.10	13	13.70	
Lip	6	2.20	2	1.10	4	4.20	
Oral cavity	86	31.20	55	30.40	31	32.60	
Oropharynx	71	25.70	56	30.90	15	15.80	
Salivary	13	4.70	7	3.90	6	6.30	
Unknown primary	10	3.60	7	3.90	3	3.20	
Salivary cavity/paranasal sinuses	17	6.20	11	6.10	6	6.30	
Nasopharynx	10	3.60	4	2.20	6	6.30	

*p* values were calculated from *t* tests for continuous variables (i.e. age and time to diagnosis) and Chi2 for categorical variables. *p* values < 0.05 were considered significant

**Table 2:** Stage at diagnosis and intention to treat paradigm for all referral pathways. Early stage is stage I/II and late stage is III/IV

Stage at diagnosis				
Referral pathway	Early stage (*n* = 76)	(%)	Late stage (*n* = 200)	(%)
H&N 2WW	41	22.70	140	77.30
Urgent and routine	20	44.40	25	55.60
A&E	1	5.00	19	95.00
Other 2WW	1	20.00	4	80.00
Follow-up	12	50.00	12	50.00
Incidental finding	1	100.00	0	0.00

Intention to treat paradigm				
Referral pathway	Curative (*n* = 233)	(%)	Palliative/best supportive care (*n* = 43)	(%)
H&N 2WW	149	82.30	32	17.70
Urgent and routine	41	91.10	4	8.90
A&E	14	70.00	6	30.00
Other 2WW	5	100.00	0	0.00
Follow-up	23	95.80	1	4.20
Incidental finding	1	100.00	0	0.00

**Fig. 1:**
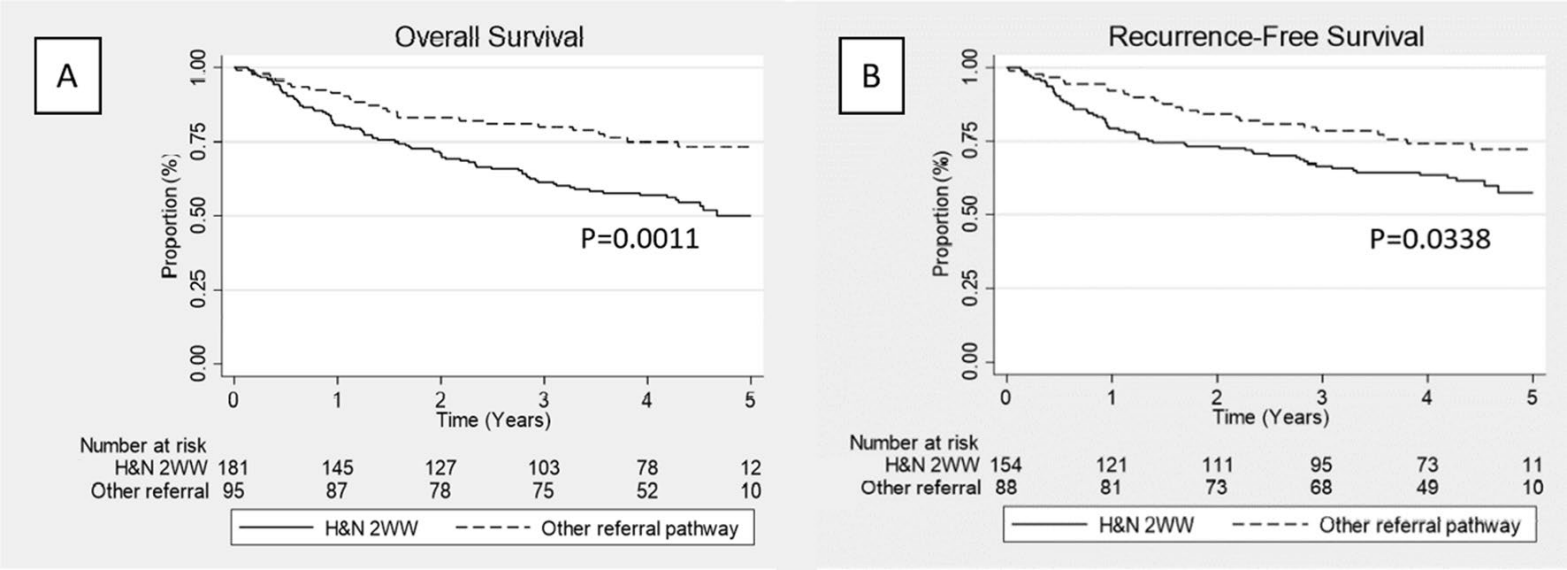
**A** Overall survival for patients under the 2WW HN referral pathway vs other referral pathways. **B** Recurrence free survival for patients under the 2WW HN referral pathway vs other referral pathways. (HN: Head and neck; 2WW: 2 weeks wait)

**Fig. 2:**
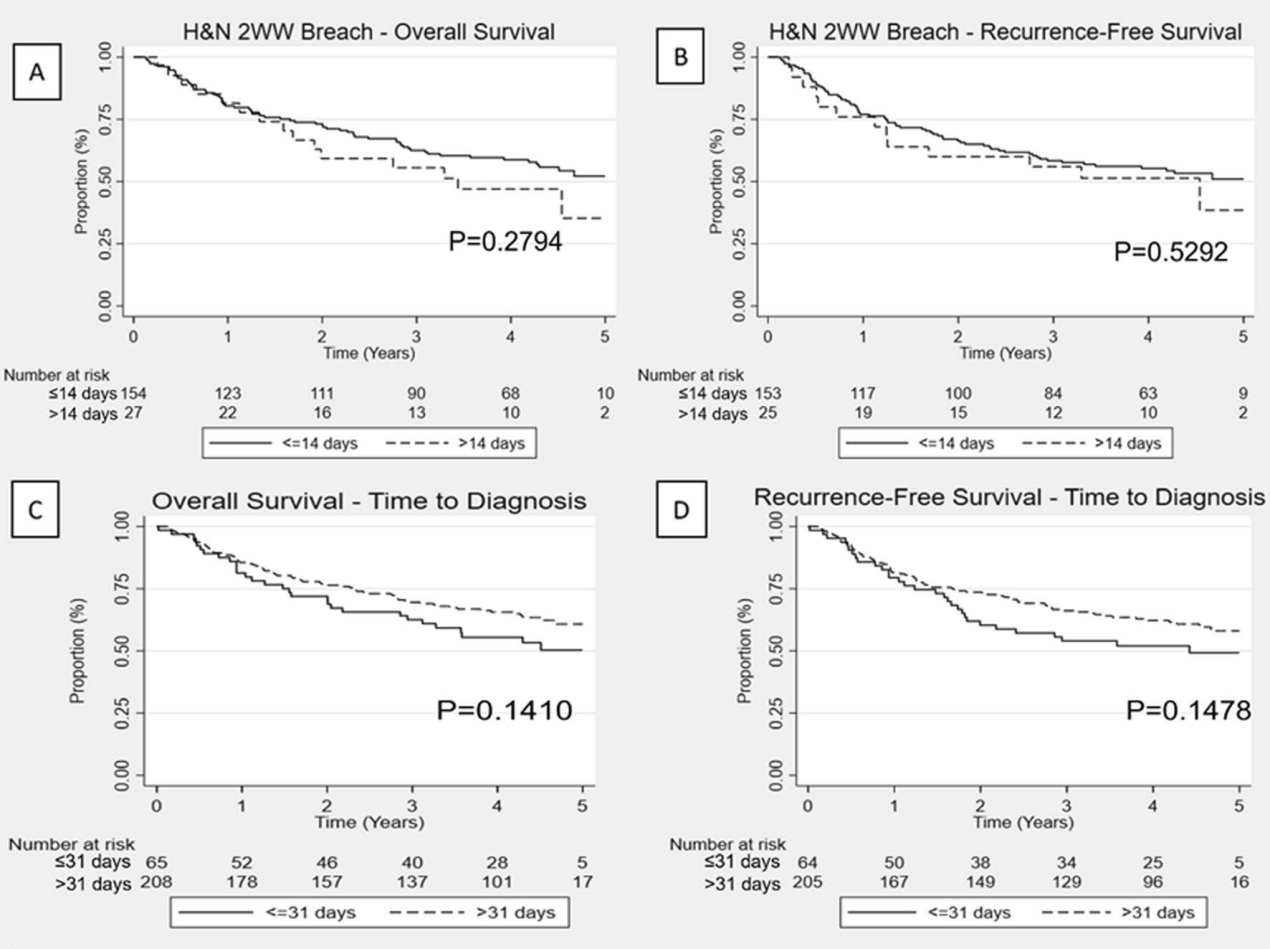
**A** Overall survival for patients under the 2WW HN referral pathway seen within 14 days from referral vs after 14 days from referral. **B** for patients under the 2WW HN referral pathway seen within 14 days from referral vs after 14 days from referral. **C** Overall survival for patients under the 2WW HN referral pathway diagnosed within 31 days from referral vs after 31 days from referral. **D** Overall survival for patients under the 2WW HN referral pathway diagnosed within 31 days from referral vs after 31 days from referral (HN: Head and neck; 2WW: 2 weeks wait)

During the studied period, a total of 5259 new 2WW referrals were received, of which, 181 were subsequently diagnosed with HNC cancer translating into a conversion rate of 3.4% (excluding patients referred from other regions for which total number of 2WW referrals received was not available). The majority of patients diagnosed under the 2WW were seen within 2 weeks from referral (154/181; 85.1%) and only five (1.7%) patients were seen more than 4 weeks after initial referral. The mean time between referral to first appointment was significantly lower in patients going through the HNC 2WW pathway (12 days) compared to other 2WW/urgent and routine (56 days, *p* < 0.0001). There was a significant difference mean wait time from referral to diagnosis in favour of patients under the 2WW pathway (54 vs 110 days, *p* < 0.0001) but this did not translate into better survival outcomes. In fact, OS and RFS were worse for patients referred under 2WW when compared to non-2WW referral (*p* = 0.0011 and *p* = 0.0338, respectively) (Fig. 1). Among HNC patient diagnosed through the 2WW pathway, there was no statistically significant difference in the OS and RFS between patients that breached the 2 weeks before specialist appointment guideline target and those that did not (*p* = 0. 2794 and *p* = 0.5292, respectively) (Fig. 2). Equally, time to diagnosis (< 31 vs > 31 days) did not significantly impact survival (*p* = 0.1410 and *p* = 0.1478, respectively) (Fig. 2).

**Fig. 3:**
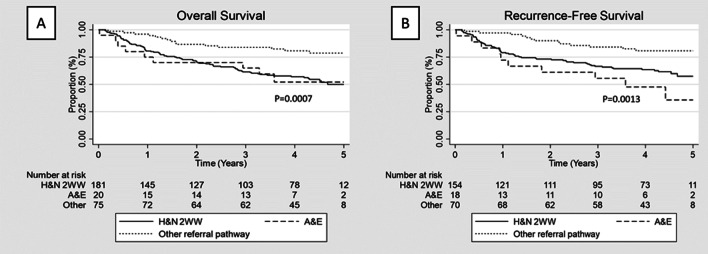
**A** Overall survival for patients under the 2WW HN referral pathway vs A&E vs other referral pathways. **B** Recurrence free survival for patients under the 2WW HN referral pathway vs A&E vs other referral pathways. (HN: Head and neck; 2WW: 2 weeks wait; A&E: accident and emergency department)

Patients diagnosed through the emergency department merit a special mention. With a total of 20 patients diagnosed during the studied period, 19 (95%) were diagnosed at an advanced stage (Table 2). Accordingly, this cohort of patients represented the largest proportion of patients treated with palliative intent (30%; *p* = 0.011) (Table 2) and the ones with worse prognosis (Fig. 3).

## Discussion

### Impact on survival and pick up rate

This study assessed a consecutive cohort of HNC patients discussed in the MDM of a large HNC network. We did not identify any survival benefit for HNC patients referred and diagnosed under the 2WW HNC pathway. In fact, patients referred through the 2WW pathway were often diagnosed at a later stage compared to those referred through other pathways and had a significantly worse OS at 5 years compared to patients diagnosed through other referral pathways. Moreover, patients who breached the 14 days from GP referral to first appointment target for the HNC 2WW pathway did not have a significantly impacted OS and RFS compared to those who were seen within the 14 days target. There is no doubt that earlier diagnosis and treatment will lead to better prognosis for the same cancer type. Accordingly, the aim of the 2WW referral pathway for HNC is to improve survival outcomes through earlier diagnosis and treatment. However, the 2WW continues to be under scrutiny as there is a lack of evidence for this system having any impact on survival and stage at diagnosis of HNC patients as shown in this study and others [9, 11]. These findings can be explained by the heterogeneous nature of HNC including not only several different subsites with characteristic presenting symptoms but also multiple histological types with varying clinical behaviour and outcomes.

Additionally, other studies have also shown that the cancer pick-up rate from 2WW referrals continues to decrease [9, 12, 13]. One of the first studies analysing the 2WW for HNC published in 2004 demonstrated that 71% of patients diagnosed with HNC were not referred using the 2WW with a conversion rate of 15% [14]. Since the publication of the aforementioned study, the number of HNC 2WW has dramatically increased and continues to do so. In our region, for the same period studied in 2017, an increase of 28.9% has been seen in 2022. In line with the increased number of referrals, our results show a larger percentage of patients diagnosed under the 2WW (65.5%) but with a decreased conversion rate (3.4%).

### Factors contributing to lack of efficacy

When analysing the apparent lack of efficacy of the 2WW pathway for HNC, several factors may be contributing such as its inappropriate usage, referral guidelines being ineffectively structured or intrinsic difficulty of suspicion and diagnosis of HNC at its early stages. With regards to inappropriate usage, high numbers of 2WW HNC referrals have been reported to not conform to the department of health guidelines [9]. GPs have also disclosed an inclination to over-refer patients under 2WW, particularly when patients are overtly pressuring [15]. Another factor might be that the 2WW referral guidelines are ineffectively structured. Tikka et al. showed that several symptoms with a high predictive value for HNC were not included in the most recent NICE HNC referral guidelines [16]. More recently, other studies have also proposed that the red flag symptoms from NICE guidelines would benefit from further review [12]. Lastly, it is also important to highlight that HNC includes several subsites and histopathologies with very varied clinical presentation, management and prognosis making clinical suspicion from non-specialists more complex.

As part of the 2WW HNC pathway, several time targets were put in place to avoid delay on treatment and theoretically improve outcomes. Those include a 14 days target for first appointment from referral and 31 days target for achieving a diagnosis. To our knowledge, this study is the first to show that there are no differences in survival between patients seen within 14 days from referral and those seen later or between patients diagnosed within 31 days from referral and those diagnosed later. These results show that although it is correct to instigate measures in order to expedite the process from referral to diagnosis and ultimate treatment, pre-established targets do not apply for all tumour locations and stages and clinical judgement must prevail when deciding which patients should be managed more urgently. Otherwise, already constrained systems risk incurring on a waste of resources in order to fulfil those time requirements that do not translate into clinical outcome benefits. This is particularly true in HNC where several locations with different clinical profiles are included.

### Improving HNC pathway

Efforts must be centred on earlier diagnosis since advanced stage at diagnosis has been related to poor outcomes [17]. However, a large proportion of patients in this study presented at late stage with this ratio being even higher among patients diagnosed within the 2WW HNC pathway. As this study and many other have found that there was no association between 2WW referrals and an early stage at diagnosis, it may be more efficient to target other causes for delay other than GP referral to the specialist appointment wait time.

Andersen et al. presented a General Model of Total Patient Delay (‘the Andersen Model’) which could be applied to a variety of disorders [18]. This model creates a framework by which we can compartmentalise and analyse each of the stages of a cancer pathway. Summarising, there are five stages defined as: ‘Appraisal delay’: time a person takes to evaluate a symptom as a sign of illness; ‘illness delay’: time from the first sign of illness until deciding to seek professional medical care, ‘behavioural delay’: time between deciding an illness requires medical care and deciding to act on this decision; ‘scheduling delay’: time between deciding to act on the decision to seek help and attending an appointment; and ‘treatment delay’: time between the first appointment and the onset of treatment. Using this model, the 2WW pathway focuses only on part of the scheduling delay (only time from GP referral to specialist but no time to GP appointment) and the treatment delay. With the reported results showing the ineffectiveness of the 2WW, targeting the first part of the pathway seems the most efficient way to improve outcomes.

Due to the nature of HNC, many tumours are asymptomatic for long periods. Further, many early cancer symptoms are non-specific. This increases the delay as patients may not become aware of their symptoms until they are at a late stage. For example, Brouha et al. reported that appraisal delay was longer among patients with pharyngeal cancer whose first symptoms were a sore throat and shorter in those with dysphagia or a neck mass [19]. Current UK government policy supports raising public awareness of cancer symptoms, encouraging people to seek help earlier for these symptoms, and increasing the evidence base around diagnostic delay with the aim of improving clinical outcomes. Studies have shown that awareness within the general population surrounding HNC is low, but a large proportion is interested in learning more [20]. Public health campaigns do have efficacy and the public receptiveness to education on HNC makes education campaigns a potential powerful tool in targeting appraisal, illness and behavioural delays and improving outcomes. For example, after an Irish mouth cancer awareness campaign, there was a subsequent increase in referral rate [21] and others have concluded that raising awareness of HNC symptoms in GPs may reduce diagnostic delay [22]. There is also a delay of GPs in symptom recognition and referral [23]. Another possible route to reduce this delay could be offering more educational resources about HNC to allied healthcare professionals, nurse practitioners and healthcare assistants [24]. Ensuring that these individuals are thoroughly educated on the presentation of HNC will target a different demographic of patients who may not attend their GP surgery regularly.

### Handling the increased number of referrals

Increasing awareness and retailoring referral indications might translate into a higher volume of 2WW HNC and a lower cancer conversion rate [13]. With a lower conversion rate, a smaller proportion of patients referred through 2WW will have a cancer diagnosis [25]. Larger numbers of non-cancer patients presenting at HNC centres affects waiting times, taxes department resources and ultimately impacts patient care. To tackle this increase in referrals, several options have showed to be effective such as telephone triaging [26] or consultations [27], the implementation of risk stratification systems to assess the risk of cancer [28] or allied health care professionals such as speech and language therapists assessing new patients [29]. This last option is particularly relevant considering that patients referred under the 2WW HNC have a higher risk of cancer in the future [30] and as seen in our series, a higher prevalence of smoking becoming this referral a great opportunity for smoking cessation referral and prevention of cancer, proven to be more effective within the 2WW than elsewhere [31].

The example from the Danish Head and Neck fast-track program is particularly interesting [32]. With similar trigger for implementation as the 2WW pathway in the UK (worse survival outcomes compared to other similar healthcare systems at time of implementation), a fast-track program for cancer patients was implemented in 2007. When suspicion for cancer arises according to a set of “red flag symptoms” being present, referral is made for urgent ENT evaluation the same or following day. This first specialist evaluation is made by a private ENT specialist and only when suspicion is confirmed, referral is made to a tertiary centre where patients are investigated within 6 days. This approach dramatically shortens the pathway but this is at expense of a filter function carried by the private specialist that is possible due to the system idiosyncrasy entailing a large use of resources that makes it difficult to reproduce elsewhere. This would justify the implementation of “filters” from GP referral to tertiary centre specialist appointment as already proposed with speech and language therapists [29] or advanced nurse practitioners.

### Strengths and limitations

To the best of our knowledge, this is the largest study to assess the impact of the HNC 2WW referral in survival outcomes. Although the retrospective nature of the study may translate into biases, assessing a large HNC network (as opposed to an individual hospital) with patients discussed at the centralised MDM for the network is an example of best practice and could translate into better survival outcomes for the whole cohort of patients. By doing so, this study decreases the risk of selection bias with all consecutive new cancer diagnosed patients for the studied period included for analysis. Furthermore, as opposed to other similar studies, we did not obtain data from routine cancer registry data which tend to have a significant amount of missing information. Although information was collected for risk factors such as alcohol and tobacco consumption, information was not available on risk factors for HNC, such as HPV status or general comorbidities; these could be associated with patient diagnostic route.

The current analysis could useful as a baseline for future work in order to improve the diagnostic pathway of HNC patients. However, our results may not be extrapolated to all healthcare systems outside of England which may differ in terms of processing of diagnosis routes.

## Conclusion

This study has found that the current 2WW HNC pathway did not offer a survival advantage and was not associated with an earlier staging at diagnosis for patients at our HNC network. It is important to target the delay in referral as well as delay in treatment to prevent late-stage cancer presentation and improve outcomes. This can be done through public health education campaigns, improving the quality of the referrals through GPs and other allied health care professionals, HNC awareness and redesigning better referral criteria to increase the conversion rate for 2WW HNC referrals. The resource for these interventions should not be underestimated.

For HNC, fulfilment of cancer time targets based on non-clinical judgement do not translate into better outcomes and although are useful to ensure smooth patients’ pathway, should not be prioritised to clinical judgement.

## Data Availability

Not applicable.
